# Calcificação Miocárdica Padrão Helicoidal de Torrent Guasp

**DOI:** 10.36660/abc.20210370

**Published:** 2022-05-04

**Authors:** Maria Marta Abraham-Foscolo, Rocío Blanco, Juan Guido Chiabrando, María Clara Llamedo, Diego Pérez de Arenaza, Mariano L Falconi

**Affiliations:** 1 Departamento de Cardiologia Hospital Italiano de Buenos Aires Buenos Aires Argentina Departamento de Cardiologia – Hospital Italiano de Buenos Aires, Buenos Aires – Argentina; 2 Departamento de Farmacologia e Toxicologia Faculdade de Medicina Universidade de Buenos Aires Buenos Aires Argentina Laboratório Aplicado de Estatística em Ciências da Saúde (LEACS), Departamento de Farmacologia e Toxicologia – Faculdade de Medicina – Universidade de Buenos Aires, Buenos Aires – Argentina

**Keywords:** Calcificação Vascular, Técnicas de Imagem Cardíaca, Insuficiência Cardíaca

## Caso clínico

Paciente do sexo masculino, 72 anos, com história pregressa de hipertensão arterial, diabetes não insulino-dependente, tabagismo ativo e angina crônica estável (angiocoronariografia mostrou estenose grave em ramo marginal obtuso de pequeno porte). Apresentava também história de etilismo, calcificação pancreática e litíase renal, sem história de Câncer, Sepse ou Tuberculose que justificassem essas calcificações difusas.

Apresentou-se no ambulatório de cardiologia com queixa de angina e dispneia classe II da New York Heart Association (NYHA). O eletrocardiograma (ECG) mostrava ritmo sinusal e sugeria hipertrofia ventricular esquerda (HVE) sem sinais de isquemia.

As imagens do ecocardiograma foram subótimas devido à janela acústica ruim, mas mostraram disfunção sistólica ventricular esquerda leve com disfunção diastólica grave (enchimento mitral restritivo não reversível foi observado), HVE e hipertensão pulmonar grave (pressão sistólica da artéria pulmonar 78 mmHg) com diâmetros de ventrículo direito normais e função sistólica. Além disso, múltiplas imagens hiperecogênicas com sombras acústicas foram observadas dentro do miocárdio, infiltrando predominantemente o septo interventricular.

Foi admitido no Hospital por sinais de insuficiência cardíaca aguda, com ortopneia, estertores pulmonares bilaterais e edema periférico bilateral, sem necessidade de oxigênio suplementar. O laboratório de soro demonstrou depuração de creatinina normal (>60 mg/ml), alto N terminal pró-peptídeo natriurético cerebral (NT-pro-BNP) e níveis séricos de troponina (6740 pg/ml e 32 pg/ml, respectivamente). Os parâmetros séricos fosfocálcicos estavam dentro da faixa normal. Notavelmente, uma equipe multidisciplinar avaliou o paciente, incluindo nefrologista, endocrinologista e cardiologistas clínicos.

**Figura 1:**
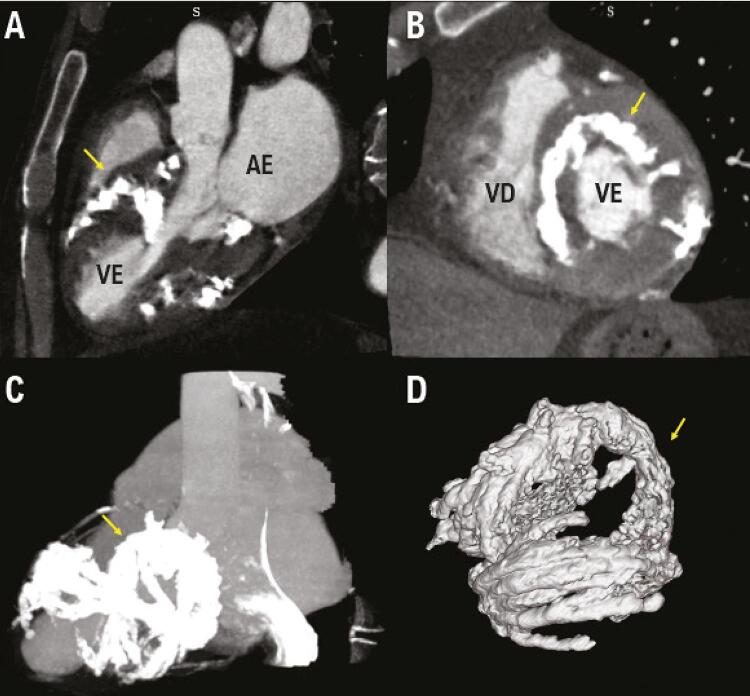
– Angiotomografia computadorizada (ATC) mostra extensa calcificação intramiocárdica (A e B). Reconstrução volumétrica na ATC mostra extensa calcificação com padrão Torrent Guasp (C e D). Os marcadores amarelos destacam a distribuição de cálcio. AE: átrio esquerdo; VE: ventrículo esquerdo; VD: ventrículo direito.

Devido aos achados anormais e subótimos no ecocardiograma, foi realizada uma angiotomografia computadorizada (ATC), que foi positiva para extensos depósitos de cálcio intramiocárdico (escore de Agatston miocárdico de 112.929) com distribuição helicoidal que se assemelhava ao padrão das fibras miocárdicas de Torrent Guasp ( Figura 1 ). O escore de cálcio da artéria coronária não foi tão alto quanto o escore de Agatston do miocárdio (escore de Agatston de 670).

**Figura 2:**
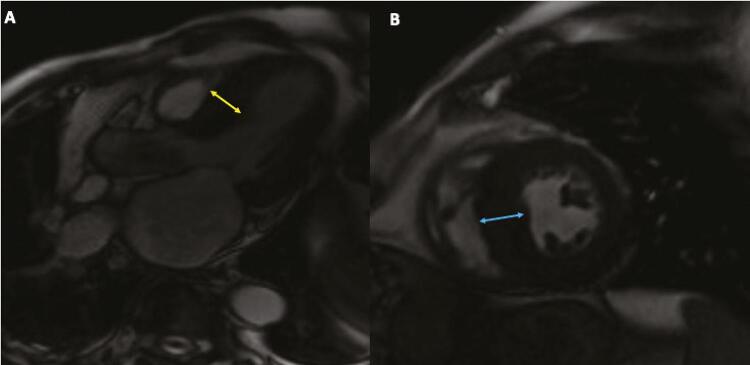
– Ressonância Magnética Cardíaca (RMC). Sequência cine mostra massa aumentada. O marcador amarelo mostra a maior largura da hipertrofia septal do VE em cinco câmaras (A). O marcador azul mostra a maior largura do VE no eixo curto (B). VE: ventrículo esquerdo.

**Figura 3:**
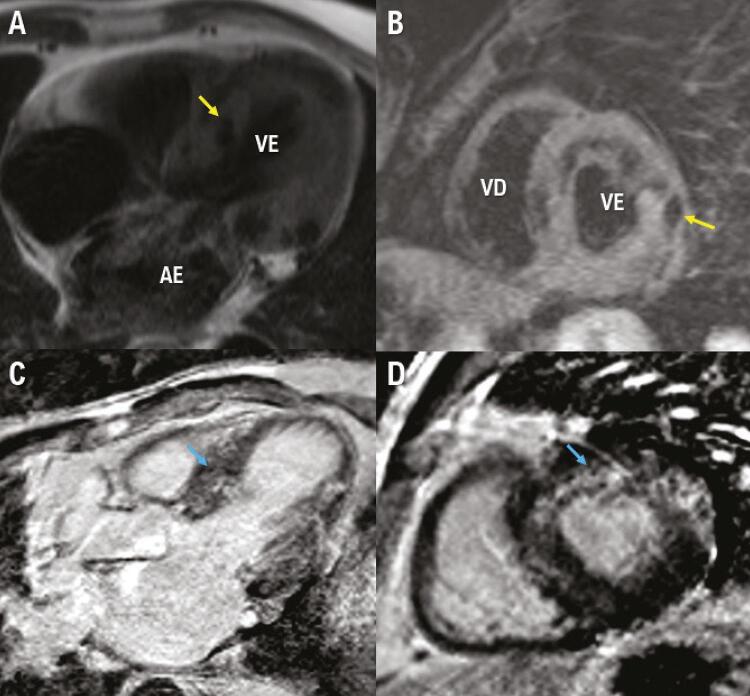
– Ressonância Magnética Cardíaca (RMC). Sequência ponderada em T1 mostra sinais de hipertrofia do ventrículo esquerdo com baixo sinal intramiocárdico compatível com cálcio (A), sequência ponderada em T2 sem edema e baixo sinal intramiocárdico (B). Intramiocárdico difuso em Realce tardio por gadolínio (LGE), sem padrão característico (C e D). Os marcadores amarelos mostram a distribuição do cálcio miocárdico. Os marcadores azuis mostram LGE miocárdico. VE: ventrículo esquerdo; VD: ventrículo direito; AE: átrio esquerdo.

Uma Ressonância Magnética Cardíaca (RMC) com contraste foi realizada para identificar melhor as características do tecido. Mostrou função sistólica do ventrículo esquerdo normal (fração de ejeção do ventrículo esquerdo de 54%), com aumento da massa miocárdica e hipocinesia basal e septal ( Figura 2 ). O ventrículo direito tinha função sistólica normal. As imagens ponderadas em T1 e T2 mostraram áreas de nulidade intramiocárdicas focais sugerindo depósitos de cálcio no miocárdio. O realce tardio com gadolínio (LGE) foi positivo para realce intramiocárdico nos segmentos basal, medial e ântero-apical, compatível com fibrose não isquêmica. O comprometimento pericárdico estava ausente ( Figura 3 ). Além disso, áreas de calcificação miocárdica na ATC correlacionaram-se com LGE ao redor dos depósitos de cálcio na RMC.

O paciente foi tratado com diuréticos de alça endovenosos e recebeu alta com melhora dos sintomas. A cintilografia com pirofosfato de 99m-tecnécio foi realizada durante o seguimento, negativa para amiloidose cardíaca. Além disso, para obtenção de material para amostragem histológica, foi realizada biópsia endomiocárdica, mostrando achados histológicos normais.

## Discussão

Os depósitos de calcificações miocárdicas podem estar presentes em múltiplos cenários fisiopatológicos, como calcificações distróficas por doença cardíaca de base e doenças sistêmicas idiopáticas ou metastáticas.^1^ Além disso, as calcificações miocárdicas geralmente representam as sequelas de dano tecidual local e necrose celular e estão associadas a um risco aumentado de eventos cardiovasculares (arritmias ventriculares e disfunção sistólica/diastólica levando à insuficiência cardíaca).^1 , 2^ O paciente apresentava história de hipertensão, diabetes e doença arterial coronariana. Frequentemente, a hipertensão não é suficiente para explicar completamente a calcificação miocárdica, sendo comumente associada à doença renal crônica (DRC), onde a calcificação está associada a um distúrbio fosfocálcico.^3^ Além disso, o diabetes produz um distúrbio inflamatório sistêmico que pode contribuir para um aumento da calcificação coronariana.^4^ Além disso, a doença arterial coronariana é uma causa muito comum de calcificação miocárdica distrófica.^1^ A cardiomiopatia hipertensiva ou infiltrativa não pode ser totalmente excluída, apesar dos achados de TC e RMC.

Esses padrões de calcificação podem ser diagnosticados com múltiplas modalidades de imagem, sendo a ATC a modalidade padrão-ouro para identificação e caracterização de calcificações miocárdicas. De fato, diferentemente do padrão de calcificação bem definido nas patologias intrínsecas do miocárdio, a infiltração de cálcio por doenças sistêmicas geralmente tem um padrão difuso. Além disso, a RMC pode fornecer mais caracterização tecidual, sugerindo calcificação miocárdica em áreas intramiocárdicas de baixo sinal e miocárdio cicatrizado ao redor de calcificações por imagens de LGE.^5 , 6^

A Teoria de Torrent Guasp foi originalmente descrita macroscopicamente em pacientes post mortem, em que as fibras miocárdicas se estruturavam como uma faixa estendida desde a raiz da artéria pulmonar até a raiz da aorta circunscrevendo os dois ventrículos em uma fibra com padrão de dupla hélice. Esse padrão é responsável pelo funcionamento normal e efetivo do coração, explicando a variação do volume intraventricular dentro de cada batimento.^7 , 8^ A pesquisa mostrou que essa distribuição de dupla hélice raramente é encontrada em modalidades de imagem não invasivas, tornando este caso uma representação viva do padrão Torrent Guasp.

## Conclusão

Apresenta-se um caso de insuficiência cardíaca com fração de ejeção preservada com calcificação miocárdica difusa e atípica seguindo a distribuição Torrent Guasp. Múltiplas causas (isto é, isquêmica, hipertensiva ou infiltrativa) podem contribuir para a origem da calcificação, a que em última análise está associada a um pior desfecho clínico. Diversas modalidades de imagem são fundamentais para alcançar um diagnóstico específico e, eventualmente, um tratamento específico.
