# Supplementary material for: Calcificação Miocárdica Padrão Helicoidal de Torrent Guasp
Source: Arq Bras Cardiol. 2022 May 4;118(5):992–5. [Article in Portuguese] doi: 10.36660/abc.20210370 (PMC9368865; doi:10.36660/abc.20210370)
Supplement: Translation

# Translation: Torrent Guasp’s Helicoid Pattern Myocardial Calcification

**Authors:** Maria Marta Abraham-Foscolo, Rocío Blanco, Juan Guido Chiabrando, María Clara Llamedo, Diego Pérez de Arenaza, Mariano L Falconi

**Affiliations:** 1 Argentina Departamento de Cardiologia – Hospital Italiano de Buenos Aires, Buenos Aires – Argentina; 2 Argentina Laboratório Aplicado de Estatística em Ciências da Saúde (LEACS), Departamento de Farmacologia e Toxicologia – Faculdade de Medicina – Universidade de Buenos Aires, Buenos Aires – Argentina

**Keywords:** Vascular Calcification, Cardiac Imaging Techniques, Heart Failure

## Clinical case

72-year-old male patient with a past medical history of arterial hypertension, non-insulin-dependent diabetes, active tobacco consumption and chronic stable angina (coronary angiography showed severe stenosis in small size obtuse marginal branch). He also had a history of alcohol consumption, pancreatic calcification and renal lithiasis, without a history of Cancer, Sepsis or Tuberculosis to justify these diffuse calcifications.

He presented to the outpatient cardiology clinic complaining of angina and dyspnea New York Heart Association (NYHA) class II. His electrocardiogram (ECG) showed sinus rhythm and suggested left ventricular hypertrophy (LVH) without signs of ischemia.

The echocardiogram images were suboptimal due to poor acoustic window but showed mild left ventricular systolic dysfunction with severe diastolic dysfunction (non-reversible restrictive mitral filling was seen), LVH and severe pulmonary hypertension (systolic pulmonary artery pressure 78 mmHg) with normal right ventricle diameters and systolic function. Furthermore, multiple hyperechogenic images with acoustic shadows were seen within the myocardium, predominantly infiltrating the interventricular septum.

He was admitted to the Hospital due to signs of acute heart failure, with orthopnea, bilateral pulmonary rales, and bilateral peripheral edema, without the need for supplementary oxygen. The serum laboratory demonstrated normal creatinine clearance (>60 mg/ml), high N terminal pro-brain natriuretic peptide (NT-pro-BNP) and troponin serum levels (6740 pg/ml and 32 pg/ml, respectively). Serum phospho-calcic parameters were within the normal range. Notably, a multidisciplinary team evaluated the patient, including a nephrologist, endocrinologist, and clinical cardiologists.

Due to the abnormal and suboptimal findings in the echocardiogram, a computed tomography angiography (CTA) was performed, which was positive for extensive intramyocardial calcium deposits (myocardial Agatston Score of 112929) with a helicoid distribution that resembled Torrent Guasp’s myocardial fibers pattern ( Figure 1 ). The Coronary Artery Calcium Scoring was not as high as the myocardial Agatston score (Agatston score of 670).

**Figure 1:** – Computed tomography angiography (CTA) shows extensive intramyocardial calcification (A and B). CTA volumetric reconstruction shows extensive calcification with Torrent Guasp pattern (C and D). The yellow markers highlight calcium distribution. LA: left atrium; LV: left ventricle; RV: right ventricle.

A Cardiac Magnetic Resonance (CMR) with contrast was performed to identify the tissue characteristics further. It showed normal left ventricular systolic function (left ventricular ejection fraction of 54%), with an enlarged myocardial mass and basal and septal hypokinesis ( Figure 2 ). The right ventricle had a normal systolic function. T1 and T2 weighted images showed focal intramyocardial nulling areas suggesting myocardial calcium deposits. Late gadolinium enhancement (LGE) was positive for intramyocardial enhancement in the basal, medial, and antero-apical segments, compatible with non-ischemic fibrosis. The pericardial compromise was absent ( Figure 3 ). In addition, myocardial calcification areas in CTA correlated to LGE around calcium deposits on CMR.

**Figure 2:** – Cardiac Magnetic Resonance Imaging (CMR). Cine sequence show enlarged mass. The yellow marker shows the largest width of the LV septal hypertrophy in five chambers (A). The blue marker shows the largest width of LV in the short axis (B). LV: left ventricle.

**Figure 3:** – Cardiac Magnetic Resonance Imaging (CMR). T1 weighted sequence show signs of left ventricle hypertrophy with intramyocardial low signals compatible with calcium (A), T2 weighted sequence without edema and intramyocardial low signal (B). Diffuse intramyocardial Late gadolinium enhancement (LGE), without a characteristic pattern (C and D). The yellow markers show myocardial calcium distribution. The blue markers show myocardial LGE. LV: left ventricle; RV: right ventricle; LA: left atrium.

The patient was treated with intravenous loop diuretics and was ultimately discharged with symptom improvement. 99m-technetium pyrophosphate scintigraphy was performed during the follow-up, negative for cardiac amyloidosis. Also, to obtain material for histologic sampling, an endomyocardial biopsy was performed, showing normal histology findings.

## Discussion

Myocardial calcifications deposits may be present in multiple pathophysiological scenarios, such as dystrophic calcifications due to underlying cardiac disease and idiopathic or metastatic systemic diseases.^1^ Moreover, myocardial calcifications generally represent the sequelae of local tissue damage and cellular necrosis and are associated with an increased risk of cardiovascular events (ventricular arrhythmias and systolic/diastolic dysfunction leading to heart failure).^1 , 2^ The patient presented with a history of hypertension, diabetes, and coronary artery disease. Frequently, hypertension is not enough to fully explain myocardial calcification, and it is commonly associated with chronic kidney disease (CKD), where calcification is associated with a phospho-calcic disorder.^3^ Furthermore, diabetes produces a systemic inflammatory disorder which may contribute to an increased coronary calcification.^4^ In addition, coronary artery disease is a very common cause of dystrophic myocardial calcification.^1^ Hypertensive or infiltrative cardiomyopathy cannot be fully excluded despite the CT and CMR findings.

These calcification patterns may be diagnosed with multiple imaging modalities, being the CTA the gold standard modality for identifying and characterizing myocardial calcifications. Indeed, unlike the well-defined calcification pattern in intrinsic myocardial pathologies, calcium infiltration due to systemic diseases generally has a diffuse pattern. Moreover, CMR might provide further tissue characterization by suggesting myocardial calcification in low signal intramyocardial areas and scarred myocardium surrounding calcifications by LGE images.^5 , 6^

The Torrent Guasp Theory was originally described macroscopically in post mortem patients, in which myocardial fibers were structured as an extended band from the root of the pulmonary artery to the root of the aorta circumscribing the two ventricles in a double helix pattern fiber. This pattern is responsible for the normal and effective function of the heart, explaining the variation of intraventricular volume within each beat.^7 , 8^ Research has shown that this double helix distribution is rarely found in non-invasive imaging modalities, making this case a living representation of the Torrent Guasp pattern.

## Conclusion

A case of heart failure with preserved ejection fraction with a diffuse and atypical myocardial calcification following the Torrent Guasp distribution is presented. Multiple causes (i.e., ischemic, hypertensive, or infiltrative) may contribute to the origin of the calcification, which is ultimately associated with a worse clinical outcome. Diverse imaging modalities are fundamental to achieving a specific diagnosis and eventually a specific treatment.
